# Expression of Ki-67 in Oral Lichen Planus: A Systematic Review and Meta-Analysis

**DOI:** 10.30476/dentjods.2023.98491.2082

**Published:** 2024-12-01

**Authors:** Aila Bahramian, Paria Motahari, Alireza Hanifenezhad

**Affiliations:** 1 Dept. of Oral and Maxillofacial Medicine, Faculty of Dentistry, Tabriz University of Medical Sciences, Tabriz, Iran; 2 Dental Student, Student Research Committee, Tabriz University of Medical Sciences, Tabriz, Iran

**Keywords:** Ki-67 antigen, Lichen planus, Oral, Prognosis

## Abstract

**Statement of the Problem:**

One of the main signs of cancer development is increasing of cell proliferation activity. Expression of the Ki-67 as a cell proliferation marker is extensively utilized in pathology studies as an indicator of proliferation in human tumors. According to studies, Ki-67 plays an effective role in the pathology of malignant and pre-malignant oral mucosa lesions.

**Purpose:**

The current study aimed to systematically review the Ki-67 expression in oral lichen planus without dysplasia and compare it with oral epithelial dysplasia.

**Materials and Method:**

In this meta-analysis, all articles in the English language were searched in databases including Web of Science, PubMed, Embase, Scopus, and Google Scholar until July 2023. MeSH terms and free keywords were used in the search step. Expression of Ki-67 in oral lichen planus and oral epithelial dysplasia was analyzed by Comprehensive Meta-Analysis software.

**Results:**

Nine hundred and two articles related to the searched words were found. According to the selection criteria, 12 retrospective articles were selected. Low quality was not observed in any of the records by the Newcastle-Ottawa scale and most of them had a relatively good quality. Totally, 593 patients were examined. The heterogeneity between studies was not significant. The meta-analysis results indicated a significantly lower Ki-67 expression in oral lichen planus without dysplasia in comparison to oral epithelial dysplasia.

**Conclusion:**

The more intense expression level of Ki-67 in oral epithelial dysplasia compared with oral lichen planus was observed. The ki-67 expression could be utilized to indicate the existence and intensity of epithelial dysplasia and disease progression.

## Introduction

Oral lichen planus (OLP) is defined by an immune response involving T cells, as well as a combination of increased cell apoptosis and proliferation occurring simultaneously [ 1 - 2 ]. The lesions have a bilateral appearance and it is usually seen as a combination of different clinical subgroups. Grey or white lines might generate a linear or reticular pattern on the background of erythematous [ 3 - 4 ]. A multidisciplinary healthcare team conducts the disease diagnosis process through the patient history survey and physical examination. The clinical diagnosis of OLP is usually possible using investigating history, typical oral lesions, and involvement of skin or nails [ 5 ]. However, to distinguish between OLP and other chronic oral conditions that present as white or ulcerative lesions, such as reactive keratosis, epithelial dysplasia, chronic hyperplastic candidiasis, gastrointestinal diseases (such as oral Crohn's disease), discoid lupus erythematosus, and anemic conditions, the biopsy is essential [ 5 ]. OLP has been categorized by The World Health Organization as a threat of progression to malignancy and recommends the strict monitoring of OLP patients [ 6 ]. According to the previous reports, severe erosive disease, leading to atrophy and lesions needing systemic therapy, has the highest risk of transformation into malignancy [ 7 - 8 ].

Activation or inactivation of tumor suppressor genes that play a role in early carcinogenesis is important in the analysis of malignancy potential. Some of these molecular biomarkers are expressed in early malignant dysplasia lesions continuously. The inability in the clinical and morphological diagnosis of neoplasm might indicate that the lesion is in the early stage of carcinogenesis. Therefore, it could be diagnosed at the molecular level [ 9 - 10 ]. The Ki-67 which is a nuclear protein linked to cell proliferation exists in all cell cycle phases, but it is not found in quiescent cells. The Ki-67 is an exceptional marker that can be used to assess the proliferation of a specific population of cells. The proportion of Ki-67-positive tumor cells is frequently associated with the clinical progression to cancer [ 11 - 12 ].

According to studies that have compared Ki-67 rate in OLP and healthy tissue, the expression of this marker in OLP is considerably higher than in healthy tissue [ 13 - 15 ]. Different results have been reported from the comparison of Ki-67 expression between OLP and oral epithelial dysplasia (OED). Some research works have shown that Ki-67 is an effective factor in the oral pre-malignant and malignant lesions progression. Therefore, Ki-67 plays the role of indicator for the prediction of OLP status. It was shown that the Ki-67 higher expression is associated with the high proliferation value in lichen planus lesions [ 13 ]. Pigatti *et al*. [ 14 ] demonstrated that the Ki-67 expression is a supplementary marker for proliferation in lesions with malignancy potential. The growth in the Ki-67 expression in epithelial dysplasia with a high-risk profile and various degrees of oral squamous cell carcinoma was also reported [ 15 ]. However, Mattila *et al*. [ 16 ] concluded that Ki-67 expression is not associated with histological parameters of tissue samples of OLP. Considering the different reports, the current study aims to examine the expression of Ki-67 in OLP prognosis by a systematic reviewing method. 

## Materials and Method

The current systematic review has been conducted as a meta-analysis with consideration of the PRISMA guideline. The registration of the review protocol was completed on the PROSPERO database with registration ID: CRD42023388413. The presentation of its research question was conducted based on the patient, intervention, comparison, and outcome in the PICO framework. The main question of this study was “Is there any variation in the expression of the Ki-67 marker in biopsied samples from OLP without dysplasia with OED?”

### Search strategy

In this paper, all articles in the English language were searched in databases, including Scopus, Web of Science, PubMed, Embase, and Google Scholar until July 2023. In addition to the mentioned databases, the search of reference list of the selected studies and related conferences were done manually. MeSH keywords, via “OR” and “AND” operators and their combination, were used to collect data. The keywords were ("oral lichen planus" OR "oral precancerous lesion(s)" OR "oral premalignant lesion(s) AND ("Ki76" OR "Ki76 Proliferation Index" OR "Ki76 antigen" OR "Ki76 index") AND ("oral epithelial dysplasia ").

### Study selection

Articles, after extraction from databases, were screened in three steps by two specialists. In the first step, according to eligibility criteria, the titles and abstracts were evaluated by two independent reviewers. The disagreements were settled down by discussion with the third one. The full text of the selected articles was investigated in the next step. 

### Eligibility criteria

Inclusion criteria were defined as (1) the cross-sectional, case-control, and cohort studies, (2) studies in which OLP was confirmed histopathologically, (3) studies that reported ki-67 protein expression with a known grading protocol and (4) studies in which the ki-67 expression were compared between dysplastic tissues and OLP.

Exclusion criteria were defined as (1) studies that included potentially malignant oral lesions other than OLP, (2) studies that included patients with only clinical symptoms of oral lichenoid lesions, (3) research works published in non-English languages, (4) studies with animal or biological samples or tissues instead of human tissue, (5) systematic reviews, case reports, and duplicate publications, and (6) low-quality articles according to Newcastle-Ottawa scale (NOS) checklist.

### Quality assessment

NOS checklist has been applied to quality checking of the selected articles and the bias risk of studies (selection, performance, diagnosis, publication bias, and attrition) was evaluated. The NOS checklist for the quality of articles includes 9 criteria in 3 total indices (selection, comparability, and outcome) containing yes or no answers and a final score of 9. A score lower than 5, a score of 5-7, and a score higher than 7 stand for the low quality, relatively good quality, and high quality of studies, respectively [ 17 ]. 

### Data extraction

The data extracted from each study was organized using Microsoft Excel software. The information obtained included the first author; published year, studied groups, sample size, Ki-67 marker expression rate, and the results of studies. The text data were analyzed manually and a significant difference in the Ki-67 marker expression rate in OLP and OED lesions was assessed.

### Statistical analysis

Considering the acceptable rate of heterogeneity, the meta-analysis of data was conducted by Comprehensive Meta-Analysis software version 2 using the fixed model. The Forest plots were used to present the results. To assess the lack of heterogeneity between studies, the I2 index and Cochran's Q statistic were used. A significance level of less than 0.05 was used to determine statistical significance. The research project received ethical approval from the Regional Ethics Committee of Tabriz University of Medical Sciences in Tabriz, Iran (The code of ethics is IR.TBZMED.REC.1401.267).

## Results

### Study selection

**Figure 1:**
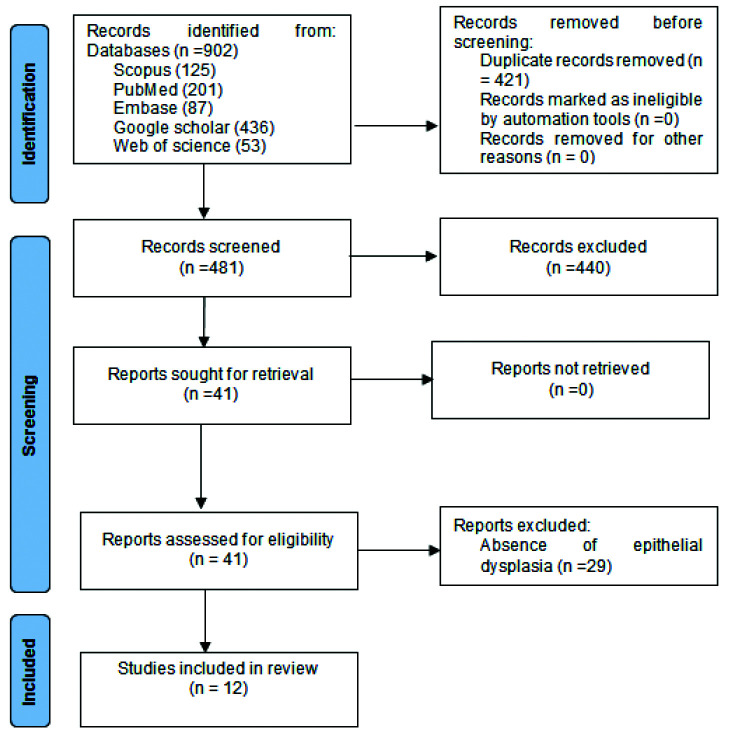
PRISMA chart of the study selection

902 articles related to the searched words were identified through the abstract in the review search. 421 articles, due to the removal of duplicates, and 440 articles, due to non-compliance with the inclusion criteria were removed. 41 articles remained in which 29 articles were removed due to the absence of epithelial dysplasia group, and 12 articles were included in the study finally [ 14 - 15 , 18 - 27 ]. The PRISMA diagram indicates the process of articles selection (Figure 1). The information resulting from the survey of articles has been presented in Table 1. 

**Table 1:** The information on reviewed articles

Authors	Study group	Number	Sex	Age	The number of Ki-67 positive cells (%)	*p* Value	Results
Garcia-PolaVallejo *et al*. [ 18 ]	OLP without dysplasia	10	6F,4M	58	14%	*p*< 0.05	The ki-67 antigen is a nuclear proliferation marker that allows for distinguishing proliferative differences between OLP and OED.
	OED	10	3F/7M	57	79%		
Girod *et al*. [ 19 ]	OLP without dysplasia	23	-	-	10.7%	*p*> 0.05	Patients with dysplasia had a relatively high proliferative activity (Ki-67 expression) compared to OLP.
	OED	34	-	-	14.2%		
Idrees *et al*. [ 20 ]	OLP without dysplasia	34	-	65.78±11.1	20.28%	*p*< 0.05	Cytomorphological evaluation in distinguishing between OLP and OED has an accuracy of 77.27%. Cellular blocks obtained from oral liquid-based brush cytology with immunohistochemical staining of Ki-67 are trustable and low-degree invasive alternatives for surgery biopsies to diagnose OLP from OED.
	OED	24	-	-	70%		
Vinay Kumar *et al*. [ 15 ]	OLP without dysplasia	20	-	-	11.03%	*p*> 0.05	Ki-67 expression in OLP was lower than OED but higher than normal mucosa.
	OED	20	-	-	18.95%		
Mittal *et al*. [ 21 ]	OLP without dysplasia	4	-	46.6±9.94	20%	*p*> 0.05	Ki-67 is a proliferative marker for oral lesions progression.
	OED	35	-	-	30%		
Ono *et al*. [ 22 ]	OLP without dysplasia	83	60F, 23M	59.8	8.4%	*p*< 0.05	With the increase in the degree of dysplasia, Ki-67 expression gets more evident. So, it has great potential in distinguishing OED lesions from OLP.
	OED	72	45F, 27M	61.2	30.6%		
Pigatti *et al*. [ 14 ]	OLP without dysplasia	14	-	32-76	42%	*p*> 0.05	The Ki-67 expression could be considered a supplementary marker for proliferative activity in lesions with malignant potential.
	OED	14	-	41-73	55.7%		
Raju *et al*. [ 23 ]	OLP without dysplasia	2	-	39.5 (21-60)	17.5%	*p*> 0.05	There was more intense staining of Ki-67 in epithelial dysplasia compared with OLP, which shows its ability as a marker for the presence and severity of dysplasia.
	OED	25	-	51.1 (30-70)	33.2%		
Rosa *et al*. [ 24 ]	OLP without dysplasia	30	21F,9M	51.8	11.60%	*p*> 0.05	There was a high expression of Ki-67 in 8 out of 30 OLP cases compared to the mean of OED (14.4). Ki-67 expression levels point out that OLP specific lesion might have a moderate malignant potential and it should be followed up closely.
	OED	30	18F,12M	49.4	14.40%		
Shailaja *et al*. [ 25 ]	OLP without dysplasia	30	14M, 16F	41	% 5.6	*p*> 0.05	OLP and OED groups had a potential affinity for malignancy compared to healthy people and Ki-67 could be considered trustworthy prognosis markers for malignancy.
	OED	30	16M, 14F	41.1	7.16%		
Zargaran *et al*. [ 26 ]	OLP without dysplasia	16	15 F,1M	38.37±12.77	13.88 %	*p*> 0.05	The malignant transformation potential for OLP could not be considered definitive. Ki-67 expression in OLP had no significant difference with mild epithelial dysplasia.
	OED	20	9 F,11M	57.65±12.03	19.16 %		
Sanketh *et al*. [ 27 ]	OLP without dysplasia	7	4 F,3M	<40,2;>40,5	57.1%	*p*> 0.05	The ki-67 expression between OLP and OED had no statistically significant difference.
	OED	6	6 M	<40,1; >40,5	100%,		

Oral epithelial dysplasia lesions (OED), oral lichen planus (OLP)

### Study characteristics

12 retrospective studies, which had been conducted on the paraffin blocks, were entered into this meta-analysis. The sample volume of these studies ranges from 13 to 155 individuals in the age group of above 35 years old. The total number of studied patients was 593. 273 patients affected by OLP without dysplasia and 320 patients affected by OED were in the compared groups. Both genders of men and women were studied. 

### Quality assessment

The quality of observational studies entered into the meta-analysis was surveyed using the NOS checklist. 11 studies had a relatively good quality and one study had a good quality (Table 2).

**Table 2:** The quality of studies using Newcastle-Ottawa scale (NOS) criterion

Authors and publication year	Selection				Comparability		Outcome			Total score	Quality z
	Case definition adequate	Representativeness of the cases	Selection of controls	Definition of controls	Main factor*	Additional factor^y^	Ascertainment of exposure	Same method of ascertainment for cases and controls	Nonresponse rate		
Garcia-Pola Vallejo *et al*. [ 18 ] 2001	+	+	+	+	-	-	+	+	-	6	Fair
Girod *et al*. [ 19 ] 1998	+	+	-	+	-	-	+	+	-	5	Fair
Idrees *et al*. [ 20 ] 2022	+	+	-	-	+	+	+	+	-	6	Fair
Vinay Kumar *et al*. [ 15 ] 2015	+	+	+	+	-	-	+	+	-	6	Fair
Mittal *et al*. [ 21 ] 2022	+	+	-	-	+	-	+	+	-	5	Fair
Ono *et al*. [ 22 ] 2021	+	+	-	-	+	+	+	+	-	6	Fair
Pigatti *et al*. [ 14 ] 2015	+	+	+	+	-	-	+	+	-	6	Fair
Raju *et al*. [ 23 ] 2005	+	+	-	-	+	+	+	+	-	6	Fair
Rosa *et al*. [ 24 ] 2018	+	+	-	-	+	+	+	+	-	6	Fair
Shailaja *et al*. [ 25 ] 2014	+	+	+	+	+	+	+	+	-	8	Good
Zargaran *et al*. [ 26 ] 2013	+	+	-	-	+	+	+	+	-	6	Fair
Sanketh *et al*. [ 27 ] 2019	+	+	-	-	+	+	+	+	-	6	Fair

* : Age was matched between two groups.

y : Sex was matched between two groups.

z : Good quality (score: >7) and fair quality (score: 5-7), Low quality (score: <5).

### Meta-analysis

**Figure 2:**
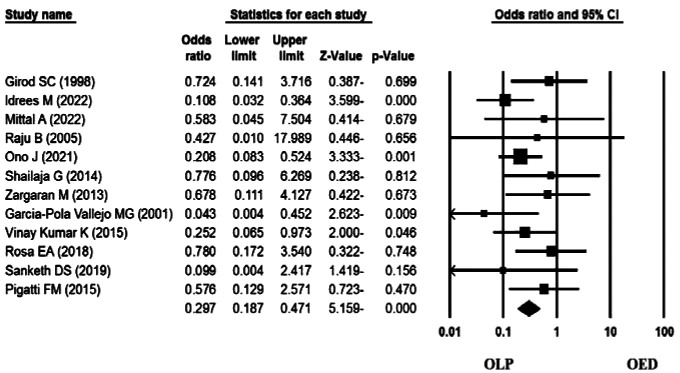
The resulting forest plot of the statistical data meta-analysis

Significant heterogeneity was not observed for the selected cases (Q-value= 2.66, df= 3, I^2^= 0.00, *p* value= 0.45). The meta-analysis results and Figure 2 show that Ki-67 expression in OLP without dysplasia was lower than in epithelial dysplasia lesions. This rate was statistically significant (Odds Ratio=0.297, 95% Confidence Interval=0.18–0.47, *p* value< 0.001). In addition, the results of studies show that with the increase in the degree of dysplasia, Ki-67 expression gets more evident. Therefore, it has great potential in distinguishing OED lesions from OLP.

## Discussion

The Ki-67 expression has been studied in patients affected by OLP and epithelial dysplasia in this systematic review for the first time. The quality of selected articles was evaluated through the NOS checklist. According to the results of this assessment, a score of 6 was acquired for a total mean of the quality of articles. None of the studies had low quality and most studies had an acceptable quality. The lower expression of Ki-67 in OLP compared to epithelial dysplasia lesions was reported in this meta-analysis (Odds Ratio= 0.29, 95% Confidence Interval=0.18–0.47, *p* value< 0.001).

The OLP is characterized by dense T lymphocytes infiltration in the sub-epithelial region and degeneration of basal keratinocytes histologically. Although this lesion, especially its erosive type, is considered premalignant in some cases, its molecular profile is considered more similar to the normal epithelium than the dysplastic one in other cases [ 1 - 5 ]. Various genetic mutations are involved in the changing of normal epithelium to neoplastic one, which destroys the mechanism of apoptosis control followed, by change in cell differentiation. The increase in mitotic activity changes the mature pattern of epithelial cells [ 11 , 28 ]. The change in the cell proliferation mechanism and apoptosis results in carcinogenesis. In other words, the higher capacity of cell proliferation could potentially be an initial indicator of the development of cancer and a crucial factor in the progression of the disease [ 10 ]. The change in the creation and function of proteins associated with these fields might act as a marker in the transformation to malignancy. The Ki-67 is a marker for cell proliferation in the perichromosomal region and its expression is strongly linked to cell proliferation and is commonly utilized in pathology researches as the proliferation indicator for measurement of the proportion of growing cells in human tumors [ 12 , 29 ]. 

60-90 minutes has been estimated as the half-life of the Ki-67 antigen. At first the Ki-67 antigen is expressed in the S phase then it gradually increases through S and G2 phases. After cell division, cells enter the G1 phase with a high rate of this antigen and a fast decrease of its amount occurs in this step [ 11 - 12 ].

According to studies, Ki-67 expression is associated with higher degrees of OED may occur in the early stages of oral cancer development [ 30 - 32 ]. 

Dysplasia is a change starting in the basal and para-basal parts of the epithelium. These abnormal changes are able to involve the whole thickness of epithelium [ 33 - 34 ]. Ki-67 is present in the basal layer's second row in normal epithelium, but in epithelial dysplasia, Ki-67 indicates the accumulative expression from the basal/parabasal layer to the spinous layer [ 15 ]. According to the Ono *et al*. [ 22 ], accumulative expression of Ki-67 gets more evident with an increase in the dysplasia degree. So, it is useful in the diagnosis of dysplasia lesions from OLP without dysplasia. 

In the study by Idrees *et al*. [ 20 ] cytomorphological evaluation was associated with 77.27% accuracy in differentiating between OLP/oral lichenoid lesion and OED, while a diagnostic index using a Ki-67 based model was 100% accurate in distinguishing cases of lichenoid lesion with OED. The results of Sanketh *et al*. [ 27 ] showed 57.1% and 100% expression of Ki-67 for lichen planus OLP and OED, respectively. According to the study of Vallejo *et al*. [ 18 ], the gene expression in dysplastic lesions was significantly more than OLP. Some studies have introduced the Ki-67 gene as a supplementary marker of cell proliferative activity in lesions with malignancy potential [ 14 , 23 ]. Mittal *et al*. [ 21 ] study also indicated that Ki-67 expression increases with disease progression from potentially malignant lesions to malignant ones.

The excessive presence of Ki-67 results in the loss of cell cycle regulation and a rise in cell proliferation rate. This secondary increase in cell proliferation is associated with damage to keratinocytes and an increase in the inflammation degree of the lesion [ 35 - 36 ]. The damaged cells in OLP target some complicated mechanisms at the molecular level that arrest the progression of the cell cycle (for repairing damaged DNA and maintaining the stability of genomic) or activate the apoptosis pathway (to destroy cells with damaged DNA). Besides, these damaged cells could enter the cell cycle and get amplified [ 37 ]. Despite the fact that the specific mechanisms responsible for the increase in cell proliferation have not yet been determined, it is possible that one of the reasons could be associated with the secretion of cytokines and inflammatory substances from damaged keratinocytes or cells involved in inflammation in OLP [ 38 ]. The findings of a study by Zargaran *et al*. [ 26 ] indicated a high rate of Ki-67 expression in OLP in comparison with epithelial hyperplasia and it also did not show a significant increase compared to mild epithelial dysplasia. Therefore, to diagnose the potential subtle changes in early steps, the OLP lesions should be followed up carefully and routinely.

Due to the lack of a means for definite diagnosis of OLP from epithelial dysplasia, biopsy and immunofluorescence techniques should be applied [ 6 ]. So, considering the results of this meta-analysis, the Ki-67 gene could be used in the differentiation of OLP without dysplasia from lesions with OED.

## Conclusion

A more intense expression of Ki-67 in OED compared to OLP was observed. Moreover, the results of studies showed that with the increase in the degree of dysplasia, Ki-67 expression gets more evident. Therefore, it has great potential to distinguish OED lesions from OLP. The Ki-67 expression could be considered as a proliferative marker for indicating the existence and intensity of epithelial dysplasia and disease progression. Finding such markers can identify lesions prone to malignancy and initiate appropriate and early treatment to prevent disease complications, as well as help improve the life quality of these patients. Therefore, this marker may be useful in the early detection and grading of oral dysplasia and be used in cancer prevention programs.
